# Supplementary material for: Empirical methods for controlling false positives and estimating confidence in ChIP-Seq peaks
Source: BMC Bioinformatics. 2008 Dec 5;9:523. doi: 10.1186/1471-2105-9-523 (PMC2628906; doi:10.1186/1471-2105-9-523)
Supplement: Additional File 1 — A variety of html documents from the USeq web site detailing the available applications, their best usage, output file type descriptions, command line menus, etc. [file 1471-2105-9-523-S1.zip › USeqUserGuides/JavaDoc.zip!/JavaDoc/bioroot/antibody/AntibodyBean.html]

AntibodyBean


|  |  |  |
| --- | --- | --- |
| **Overview** \| **Package** \| **Class** \| **Use** \| **Tree** \| **Deprecated** \| **Index** \| **Help** | |  |
| **PREV CLASS**   **NEXT CLASS** | **FRAMES**    **NO FRAMES**     **All Classes** |  |
| SUMMARY: NESTED \| FIELD \| CONSTR \| METHOD | DETAIL: FIELD \| CONSTR \| METHOD |  |


---


## bioroot.antibody Class AntibodyBean

```
java.lang.Object
  bioroot.ReagentBean
      bioroot.antibody.AntibodyBean
```

**All Implemented Interfaces:**: java.lang.Comparable

---

``` public class AntibodyBean extends ReagentBean ```

**Author:**
:   nix
    Container for holding base Antibody information, AntibodyPreps have most of it.

---

**Field Summary**

**Fields inherited from class bioroot.ReagentBean**

`log`


**Constructor Summary**

`AntibodyBean()`

`AntibodyBean(int id,
DBUtil bioRoot)`
  
          Assumes id is not zero

`AntibodyBean(java.sql.ResultSet results)`


| **Method Summary** | |
| --- | --- |
| `int` | `compareTo(java.lang.Object obj)` |
| `AntibodyPrep[]` | `getAntibodyPreps(DBUtil bioRoot)` |
| `java.lang.String` | `getAntigen()` |
| `java.lang.String` | `getFileName()` |
| `java.lang.String` | `getGeneId(DBUtil bioRoot)` |
| `java.lang.String` | `getGeneName(DBUtil bioRoot)` |
| `java.lang.String` | `getHotLinkedPlasmids(DBUtil bioRoot)`             Returns HTML plasmid names for display. |
| `java.lang.String` | `getHotLinkedStrains(DBUtil bioRoot)`             Returns HTML strain names for display. |
| `int` | `getNumberPrepUseLines()` |
| `java.lang.String` | `getOldFileName()` |
| `java.lang.String` | `getPlainTextPlasmid()`             Returns plain text of plasmid ids for archive. |
| `java.lang.String` | `getPlainTextStrain()`             Returns plain text of strain ids for archive. |
| `java.lang.String[]` | `getPlasmidConcats(DBUtil bioRoot)`             Returns an array of id: plasmidName, or empty String[]{""} if there are none. |
| `java.lang.String` | `getSource()` |
| `java.lang.String[]` | `getStrainConcats(DBUtil bioRoot)`             Returns an array of id: strainName, or empty String[]{""} if there are none. |
| `java.lang.String` | `getType()` |
| `boolean` | `isAntibodyNameUnique(DBUtil bioRoot)`             checks to see if antibody name is unique within labgroup, assumes a connection has been made |
| `boolean` | `isNameChanged()` |
| `void` | `loadAntibodyBean(java.sql.ResultSet results)` |
| `boolean` | `saveNewAntibodyPreps(DBUtil bioRoot)` |
| `boolean` | `savePlasmidIds(DBUtil bioRoot, boolean deleteOld)`             Can delete old plasmidIds from AntibodyPlasmid table, and add new entries if any are present in the plasmidConcats. |
| `boolean` | `saveStrainIds(DBUtil bioRoot, boolean deleteOld)`             Can delete old strainIds from AntibodyStrain table, and add new entries if any are present in the strainConcats. |
| `void` | `setAntibodyPreps(AntibodyPrep[] antibodyPrepBeans)` |
| `void` | `setAntigen(java.lang.String antigen)` |
| `void` | `setFileName(java.lang.String fileName)` |
| `void` | `setGeneId(int i)` |
| `void` | `setGeneName(java.lang.String geneName)` |
| `void` | `setNameChanged(boolean nameChanged)` |
| `void` | `setNumberPrepUseLines(int numberPrepsUses)` |
| `void` | `setOldFileName(java.lang.String oldFileName)` |
| `void` | `setPlasmidConcats(java.lang.String[] plasmidConcats)` |
| `void` | `setSource(java.lang.String source)` |
| `void` | `setStrainConcats(java.lang.String[] strainConcats)` |
| `void` | `setType(java.lang.String type)` |
| `boolean` | `submitNew(DBUtil bioRoot)`             Makes a new Antibody table entry, don't forget to save associated strains and plasmids after updating this bean with its new id number. |
| `boolean` | `updateOld(DBUtil bioRoot, UserBean submitter)`             Updates a Antibody entry, don't forget to update geneId |

**Methods inherited from class bioroot.ReagentBean**

`appendMessages, canModifyAll, canModifyCommon, canView, getAlias, getAvailability, getComments, getEditHistory, getGenericVisibility, getId, getLabGroupId, getLabGroupName, getLastUser, getLocation, getMessages, getName, getNotes, getOrderBy, getOrganismId, getOrganismName, getOrganization, getOwnerId, getOwnerName, getReference, getVisibility, isComplete, isReagentNameUnique, loadReagentBean, setAlias, setAvailability, setComments, setComplete, setEditHistory, setId, setLabGroupId, setLabGroupName, setLastUser, setLocation, setMessages, setName, setNotes, setOrderBy, setOrganismId, setOrganismName, setOwnerId, setOwnerName, setReference, setVisibility, updateCommonAccessFields`

**Methods inherited from class java.lang.Object**

`equals, getClass, hashCode, notify, notifyAll, toString, wait, wait, wait`

**Constructor Detail**

### AntibodyBean

```
public AntibodyBean()
```

---


### AntibodyBean

```
public AntibodyBean(java.sql.ResultSet results)
```

---


### AntibodyBean

```
public AntibodyBean(int id,
                    DBUtil bioRoot)
```

:   Assumes id is not zero


**Method Detail**

### getAntibodyPreps

```
public AntibodyPrep[] getAntibodyPreps(DBUtil bioRoot)
```

---


### compareTo

```
public int compareTo(java.lang.Object obj)
```

:   **Specified by:**: `compareTo` in interface `java.lang.Comparable` **Overrides:**: `compareTo` in class `ReagentBean`

---


### loadAntibodyBean

```
public void loadAntibodyBean(java.sql.ResultSet results)
```

---


### getGeneName

```
public java.lang.String getGeneName(DBUtil bioRoot)
```

---


### getStrainConcats

```
public java.lang.String[] getStrainConcats(DBUtil bioRoot)
```

:   Returns an array of id: strainName, or empty String[]{""} if there are none.

---


### saveStrainIds

```
public boolean saveStrainIds(DBUtil bioRoot,
                             boolean deleteOld)
```

:   Can delete old strainIds from AntibodyStrain table, and add new entries if any are
    present in the strainConcats.

---


### getHotLinkedStrains

```
public java.lang.String getHotLinkedStrains(DBUtil bioRoot)
```

:   Returns HTML strain names for display.

---


### getPlainTextStrain

```
public java.lang.String getPlainTextStrain()
```

:   Returns plain text of strain ids for archive.

---


### getPlasmidConcats

```
public java.lang.String[] getPlasmidConcats(DBUtil bioRoot)
```

:   Returns an array of id: plasmidName, or empty String[]{""} if there are none.

---


### savePlasmidIds

```
public boolean savePlasmidIds(DBUtil bioRoot,
                              boolean deleteOld)
```

:   Can delete old plasmidIds from AntibodyPlasmid table, and add new entries if any are
    present in the plasmidConcats.

---


### getHotLinkedPlasmids

```
public java.lang.String getHotLinkedPlasmids(DBUtil bioRoot)
```

:   Returns HTML plasmid names for display.

---


### getPlainTextPlasmid

```
public java.lang.String getPlainTextPlasmid()
```

:   Returns plain text of plasmid ids for archive.

---


### submitNew

```
public boolean submitNew(DBUtil bioRoot)
```

:   Makes a new Antibody table entry, don't forget to save associated strains and plasmids after
    updating this bean with its new id number.

---


### saveNewAntibodyPreps

```
public boolean saveNewAntibodyPreps(DBUtil bioRoot)
```

---


### updateOld

```
public boolean updateOld(DBUtil bioRoot,
                         UserBean submitter)
```

:   Updates a Antibody entry, don't forget to update geneId

---


### isAntibodyNameUnique

```
public boolean isAntibodyNameUnique(DBUtil bioRoot)
```

:   checks to see if antibody name is unique within labgroup, assumes a connection has been made

---


### getGeneId

```
public java.lang.String getGeneId(DBUtil bioRoot)
```

---


### getFileName

```
public java.lang.String getFileName()
```

---


### setFileName

```
public void setFileName(java.lang.String fileName)
```

---


### setPlasmidConcats

```
public void setPlasmidConcats(java.lang.String[] plasmidConcats)
```

---


### getType

```
public java.lang.String getType()
```

---


### setGeneId

```
public void setGeneId(int i)
```

---


### setType

```
public void setType(java.lang.String type)
```

---


### getAntigen

```
public java.lang.String getAntigen()
```

---


### setAntigen

```
public void setAntigen(java.lang.String antigen)
```

---


### getSource

```
public java.lang.String getSource()
```

---


### setSource

```
public void setSource(java.lang.String source)
```

---


### setAntibodyPreps

```
public void setAntibodyPreps(AntibodyPrep[] antibodyPrepBeans)
```

---


### setGeneName

```
public void setGeneName(java.lang.String geneName)
```

---


### setStrainConcats

```
public void setStrainConcats(java.lang.String[] strainConcats)
```

---


### getOldFileName

```
public java.lang.String getOldFileName()
```

---


### setOldFileName

```
public void setOldFileName(java.lang.String oldFileName)
```

---


### isNameChanged

```
public boolean isNameChanged()
```

---


### setNameChanged

```
public void setNameChanged(boolean nameChanged)
```

---


### getNumberPrepUseLines

```
public int getNumberPrepUseLines()
```

---


### setNumberPrepUseLines

```
public void setNumberPrepUseLines(int numberPrepsUses)
```


---


|  |  |  |
| --- | --- | --- |
| **Overview** \| **Package** \| **Class** \| **Use** \| **Tree** \| **Deprecated** \| **Index** \| **Help** | |  |
| **PREV CLASS**   **NEXT CLASS** | **FRAMES**    **NO FRAMES**     **All Classes** |  |
| SUMMARY: NESTED \| FIELD \| CONSTR \| METHOD | DETAIL: FIELD \| CONSTR \| METHOD |  |


---
